# Impact Fatigue Life of Adhesively Bonded Composite-Steel Joints Enhanced with the Bi-Adhesive Technique

**DOI:** 10.3390/ma16010419

**Published:** 2023-01-02

**Authors:** Alireza Akhavan-Safar, Ghasem Eisaabadi Bozchaloei, Shahin Jalali, Reza Beygi, Majid R. Ayatollahi, Lucas F. M. da Silva

**Affiliations:** 1 Institute of Science and Innovation in Mechanical and Industrial Engineering (INEGI), Rua Dr. Roberto Frias, 4200-465 Porto, Portugal; 2 Department of Materials Engineering and Metallurgy, Faculty of Engineering, Arak University, Arak 3815688349, Iran; 3 Departamento de Engenharia Mecanica, Faculdade de Engenharia da Universidade do Porto, Rua Dr. Roberto Frias, 4200-465 Porto, Portugal; 4 Fatigue and Fracture Research Laboratory, Center of Excellence in Experimental Solid Mechanics and Dynamics, School of Mechanical Engineering, Iran University of Science and Technology, Narmak, Tehran 16846, Iran

**Keywords:** adhesive joints, bi-adhesive, impact fatigue, J-N methodology, CFRP/steel

## Abstract

One of the most common loading conditions that bonded joints experience in service is repeated impact. Despite the destructive effects of impact fatigue, the behavior of metal-composite bonded joints subjected to repeated impact loads has rarely been studied in the literature. Therefore, it is of utmost importance to pay attention to this phenomenon on the one hand and to find solutions to improve the impact fatigue life of bonded composite metal components on the other hand. Accordingly, in this study, the use of the bi-adhesive technique is proposed to improve the durability of composite-metal single-lap joints (SLJs) under impact fatigue loading conditions. J-N (energy-life) method is also used to analyze the experimental data obtained. Accordingly, in the present study, the impact fatigue behavior of single adhesive metal to composite joints was analyzed experimentally based on the J-N method and also numerically using the finite element method (FEM). By using two adhesives along a single overlap, the impact fatigue life of joints between dissimilar composite and metal joints was also analyzed experimentally. The results show that the double adhesives technique can significantly improve the impact fatigue life of the tested joints. It was also found that the optimum length ratio of the adhesives (the length covered by the ductile adhesive relative to the total overlap size) is a function of the stiffness of the joint and is more pronounced for less stiff bonded joints. A linear elastic numerical analysis was also conducted to evaluate the stress state along the bloodline of the bonded joints. Results show that the compressive peel stress made at the boundary of the two adhesives can be a possible reason behind the different results observed.

## 1. Introduction

In recent decades, the use of carbon fiber reinforced polymers (CFRP) in modern industrial structures such as airframes and vehicle bodies has increased significantly. Composite structures offer advantages over metal structures in terms of high strength, fatigue resistance, and lightweight. Because of the importance of using composites in industry, the application of adhesive to join metals to composites is inevitable. On the other hand, compared to conventional joining methods, adhesive bonding is one of the most important techniques for joining different materials (including composite materials) since adhesives offer advantages such as more even stress distribution, higher fatigue strength, energy absorption, etc. Numerous studies have dealt with the mechanical performance of bonded composite structures from different points of view [1]. One of the critical topics considered by the authors is the strength of bonded composite joints subjected to high strain rates, including impact loads. Chen et al. [2] found a 75% reduction in energy absorption for composite joints subjected to high strain rate experiments compared to the quasi-static loads due to the lower elongation at failure. Huang et al. [3] numerically and experimentally investigated the low energy bending impact damage of bonded single lap joints (SLJs) with similar (composite/composite) and dissimilar (composite/steel) substrates. They found that dissimilar joints are more susceptible to low-energy bending impact damage than similar joints. Effects of a wide range of strain rates on the impact strength of epoxy adhesives were experimentally studied by Houjou et al. [4]. In another study, Chung and Kwak [5] evaluated the shear impact strength of bonded joints by conducting some impact experiments using a piezoelectric force sensor. They proposed a test methodology that would allow them to simultaneously characterize the bond strength and shock absorption of the tested joints.

The effects of high strain rate and impact loading on the performance of bonded assemblies have been explored numerically [6,7] and experimentally [8,9,10] in several studies. Borges et al. [7] developed a numerical approach to analyze the influence of the loading rate on the mechanical response of adhesive joints using cohesive zone modeling. In this method, the mechanical properties of the adhesive are defined as a function of the loading rate. They proved that by reducing the strain rate, the fracture stress is reduced for the tested materials. Ramezani et al. [11] numerically and experimentally investigated the effects of loading rate on the failure load and failure mechanism of single lap joints made of composites and hybrid composites. The results showed that the additional layers could significantly reduce the local stresses and delamination in composites subjected to both static and high loading speeds. According to their results, the mechanical response of the adhesive layer directly affects the failure mechanisms. Due to the viscoelastic response of adhesive materials, changing the strain rate can significantly influence the stiffness, strength, and ductility of the joints.

During service, joints are often subjected to several unforeseen low-energy impacts. These loads are not capable of individually causing joint failure, but they have the potential to cause accumulative damage to the adhesive layer [12]. Despite extensive studies on the impact strength of adhesive joints, there is little published work on the effects of repeated low-energy impacts on the degradation of adhesive, mechanical properties. Experimental results have shown that low-energy cyclic impacts are more destructive than normal fatigue loads [13]. The literature shows that the fatigue endurance limit of the structures under impact fatigue is extremely lower than the normal fatigue in bonded joints. By conducting a wide range of experiments, Jalali et al. [13] showed that under impact fatigue, the endurance limit of bonded steel joints is less than 8% of the joint’s impact strength, while under normal fatigue loading, the fatigue limit is around 40–50% of the static strength of the joint [14,15]. The residual static strength of adhesive joints subjected to pre-impact loads was also experimentally obtained by Kemiklioglu et al. [16]. Their results showed that the residual strength is a direct function of impact cycles. According to the experimental data [13], two different types of damage mechanisms were observed for joints subjected to impact fatigue and quasi-static loading. For quasi-static loading, the damage usually starts at the bonding ends, and the crack then propagates along the bondline. For joints subjected to low-energy fatigue impact loading, the damage starts in the middle of the overlap. This is due to the stress waves propagating through the overlap and creating a higher stress level in the middle of the joint. The fractography analysis conducted by Jalali et al. [13] showed two different regions at the fracture surface of SLJs after impact fatigue failure. The first area corresponds to damage initiation and accumulation in the center of the overlap, and the second area corresponds to rapid crack growth taking place at the joint ends before the final failure of the joints.

Despite the more uniform stress distribution in bonded joints, compared to other mechanical joining techniques, the load transfer capacity of adhesives is significantly reduced due to the local stress concentration at the overlap ends, especially for SLJs with large overlap sizes [17,18]. The rotation of the substrates due to the momentum of the applied loads leads to a local peel stress concentration at the ends of the bondline. This uneven stress distribution along the overlap can significantly affect the durability of the joints. Authors have presented various methods, such as tapering the joint parts or using adhesive fillets at the overlap ends to reduce these edge effects. However, one of the most effective techniques to reduce stress concentration is the bi-adhesive technique, where two adhesives with different properties (one more brittle and one more ductile) are applied on a single overlap [18]. The use of a ductile adhesive at the ends of the bonded joint reduces the shear and peel stresses leading to a more uniform stress distribution along the bonded area, resulting in a higher load-bearing capacity of the joint [18]. However, it should be noted that the properties of the adhesives change depending on the loading rate, so at very high strain rates, the ductile adhesive may no longer show a ductile response [7,19]. Consequently, the damping capability of the ductile adhesive decreases at higher strain rates [20]. Accordingly, it is important to understand the behavior of bi-adhesive joints under impact loads, especially for dissimilar adhesive joints that as a common material used in lightweight and load-bearing industrial structures. The bi-adhesive technique was considered by Akhavan-Safar et al. [20] to improve the impact fatigue life of similar joints (joints with similar adherends). They experimentally investigated the impact fatigue life of bi-adhesive joints as a function of the impact energy. The authors used similar steel adherends to find length ratio effects on the joint endurance limit subjected to repeated impact loads. The results showed that the bi-adhesive technique could significantly increase the impact strength of the tested joints. It was also shown that the ductile adhesive creates a second local stress concentration along the bondline, which symmetrically reduces the stress level along the bondline.

As shown in the previous studies [18,21], the fracture strength can be significantly improved by using the bi-adhesive technique when the joints are exposed to impact and quasi-static loading conditions. However, as mentioned in a recent review article [18], despite the extensive work on bi-adhesive joints subjected to quasi-static loading conditions, the use of bi-adhesive for impact loads has rarely been considered in the literature. Moreover, these few studies are limited to impact strength and not impact fatigue. They also mainly consider joints with similar substrates, while dissimilar joints, which are common in practice, have received less attention.

Accordingly, the aim of the current study is to further explore this topic by considering the application of the bi-adhesive technique in joints with dissimilar CFRP-Steel substrates subjected to repeated low-energy impacts. The results have been analyzed based on the J-N method, where the applied impact energy (J) is plotted against the fatigue life (N) on a semi-logarithmic scale. The results are compared with similar adherends bonded joints subjected to impact fatigue. A numerical analysis is also included to show the shape of the stress waves through the tested adhesive joint. The numerical modeling also helps to find the possible relation between the adhesives’ length ratio, substrate material, and the peel stress, as a critical stress component, along the overlap.

## 2. Experimental Details

### 2.1. Material

In order to manufacture dissimilar joints, two different substrate materials were used. The metal substrate was made of steel plates with a thickness of 3 mm. Ten layers of woven carbon fibers were used to manufacture the composite adherends. The hand layup method was used to create the composite plates. LY 5052 resin as the matrix was considered, and the fabricated composites were cured in a hot press for 24 h at 23 °C followed by 4 h post-curing process at 100 °C. Table 1 gives the mechanical properties of the steel and composite materials considered for the numerical study.

In order to manufacture bi-adhesive joints, two different adhesives with different properties were used. One of them is a ductile adhesive with a maximum tensile elongation of 33%, while the considered brittle adhesive has a maximum tensile elongation of 1.4%.

As already discussed by Akhavan-Safar et al. [18], the optimum E ratio is affected by several geometrical parameters such as adhesive thickness, total overlap length, adherends elasticity moduli, etc. A wide range of E ratios has been considered by researchers, from 0.003 to 0.9 [18]. Accordingly, in this study, MEGA-POX 330 was used as the ductile adhesive, and DO-GHOLO (Ghaffari Co., Iran) was considered as the brittle adhesive with an E ratio of 0.48. In order to achieve this E ratio, the resin-to-hardener weight ratio for MEGA-POX 330 was set to 0.66, and it was 1.25 for the brittle adhesive (DO-GHOLO). The joints were cured at room temperature for one week. Bulk specimens were manufactured based on ASTM D638 and were tested in a previous study. The results are shown in Table 2.

### 2.2. SLJ Geometry

**Figure 1:**
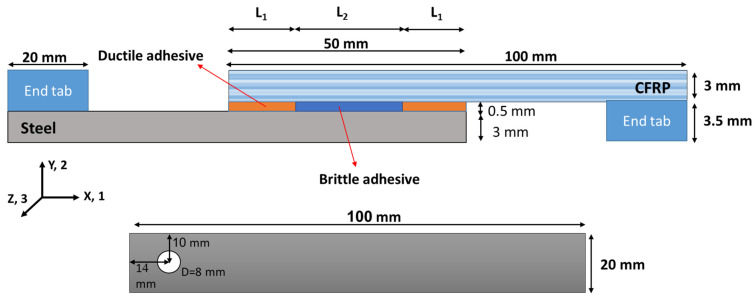
Geometry of the tested SLJs (not to scale).

Figure 1 shows the dimensions of the tested joints where a 3 mm thick steel and a 3 mm thick CFRP composite, each 100 mm long, were used as adherends to manufacture SLJs with a width of 20 mm.

As discussed in [18], the length ratio (d) in bi-adhesive joints is a key factor controlling the mechanical behavior/strength of the SLJs. The optimal d-value, which represents the highest fracture load, is a function of the joint geometry, the mechanical properties of the adhesives, and the substrate stiffness [18]. In practice, joints with different overlaps are used, which can be shorter or often longer than the joint used in the current study. In general, however, the advantages of the bi-adhesive technique are more pronounced for longer overlaps, where higher peel stress is generated at the bonding ends, limiting the strength of the joint. Consequently, 50 mm overlap length as a lab-level overlap size in SLJs was considered to be representative of real joints with similar overlap sizes that can benefit from the bi-adhesive technique and, more importantly, help readers to easier compare the current results with those previously published in [20,21], where the same overlap length has been used. However, further studies are needed to analyze size effects on the mechanical behavior of bi-adhesive joints.

Holes were made at the ends of the adherend specimens to be able to mount the specimens on the drop weight impact fatigue test machine and also to apply load on a loading pin placed in these holes.

Adhesive thickness plays a key role in joint strength [23]. Numerical studies have shown that increasing the adhesive thickness in bi-adhesive SLJs decreases the concentration of shear and peel stresses along the lap length [24] (compared to a joint with a single brittle adhesive where a higher value of peel stress is observed along the lap length [21]). Consequently, increasing the adhesive thickness causes a more uniform load transfer through the adhesive layer, resulting in an increase in the strength of bi-adhesive joints. However, similar to SLJs, there is an optimal adhesive thickness at which maximum strength is achieved [21]. Based on experimental data, the influence of adhesive thickness on ultimate load is more pronounced for joints with a lower E [25] and/or a lower aspect ratio [21]. The optimum adhesive layer thickness for bi-adhesive joints is generally assumed to be in the range of 0.4 to 0.5 mm [18]. For the tested joints, the adhesive thickness was set to 0.5 mm.

One of the most important parameters that can significantly change the strength of bi-adhesive joints is the adhesives length ratio (d). If the total overlap is defined as L, and the total length of the overlap occupied with the ductile adhesive is considered as 2L_1_ (L_1_ at each overlapping end), then the length ratio is defined as d = L_1_/L. Accordingly, the length of the overlap covered by the brittle adhesive is equal to L_2_, which is obtained as L − 2L_1_. Previous studies [18,20,21] have shown that the optimum length ratio for bi-adhesive joints is between 0.1 and 0.3 depending on various parameters, such as the stiffness ratio of the two adhesives. However, based on the definition of the length ratio, the maximum possible length ratio is 0.5, which corresponds to joints where the ductile adhesive covers the entire overlap. Accordingly, in this study, two different adhesive length ratios, including 0.1 and 0.2, were considered. Joints were also manufactured with the stiffer (brittle) adhesive, and these results were used as a reference.

### 2.3. Manufacturing

Preparing the surface of the adherends by removing contamination, dust, oxide, etc., is an essential step in manufacturing adhesive joints. Good surface treatment can significantly improve the quality of the adhesion between the adherend and the surface. One of the most common techniques used for preparing the surface of the steel is sandblasting. Accordingly, the surface of steel adherends was sandblasted, followed by acetone cleaning.

As recommended by various authors [26], smooth sandpaper followed by acetone cleaning was used to prepare the bonding surface of the composite adherends.

**Figure 2:**
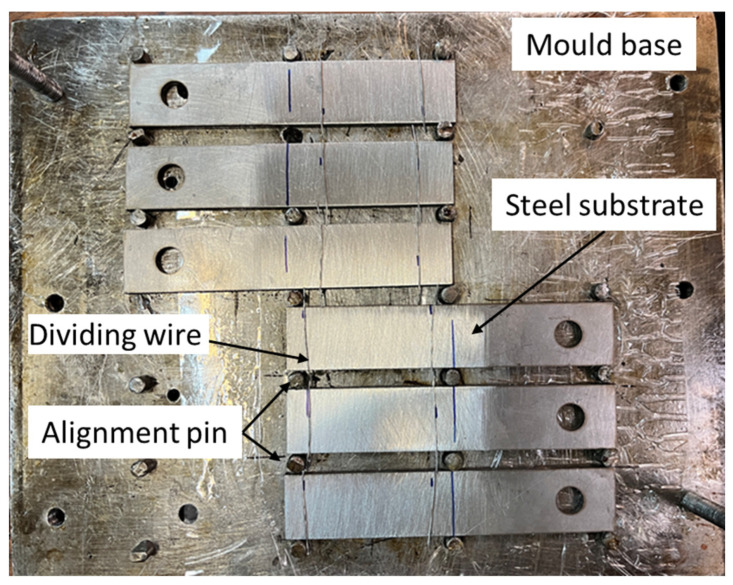
Mold used for the manufacturing of the SLJs.

One of the important steps in manufacturing bi-adhesive joints is to control the adhesive length ratio. The ductile and brittle adhesives usually have different viscosities; however, it is often difficult to control the length ratio of the adhesives without using a physical boundary between the two. Authors have already considered several techniques for controlling the length ratio. Using a physical boundary not only avoids mixing the two adhesives but also controls the adhesive thickness. However, in the current research, aluminum wires of 0.5 mm diameter (the same as the adhesive thickness) were placed at the boundary of the two adhesives to avoid mixing the two adhesives during the curing process and also to control the length ratio of the adhesives. The wire can also be seen as a useful technique to control the thickness of the adhesive. It should be noted that the presence of a wire as a common length ratio control technique can affect the overall behavior of the joints, but without a physical barrier, it is difficult to control the length ratio of the adhesives. On the other hand, due to the presence of the wires in all the tested joints, the effect of wires on the results was ignored. To manufacture the joints, first, the ductile adhesive was applied at both ends, and then the brittle adhesive was applied in the middle of the overlap. To keep the bonded adherends aligned during the curing process, a specific mold, shown in Figure 2, was employed. Bonded joints were cured at room condition for 7 days.

It should be noted that the idea behind using the bi-adhesive technique is to improve the strength of stiff joints bonded with a brittle adhesive [18]. As already shown experimentally in [21], by increasing the adhesive aspect ratio (d) above the optimal value (at which the lap is mostly or completely covered by the ductile adhesive), the length of the brittle adhesive in the middle of the lap is not reached enough to withstand the transmitted load, which of course is not an ideal condition in practice. It should also be noted that in real applications, the joints often experience a wide range of strain rates from quasi-static to impact. Consequently, only using a ductile adhesive with low stiffness along the lap cannot withstand the service loads, especially at low strain rates. Accordingly, in the current study, joints with a single ductile adhesive were not analyzed.

### 2.4. Test Procedure

For quasi-static tests, the joints were tested under tensile loading using a universal tensile testing machine. The load was measured using the machine load cell, and the displacement is the crosshead displacement measured by the test machine. In addition, to align the loading line with the bondline, end tabs were bonded to both ends of the joints (as shown in Figure 1) before testing the specimens. The rate of displacement in quasi-static tests was set to 1 mm/min.

**Figure 3:**
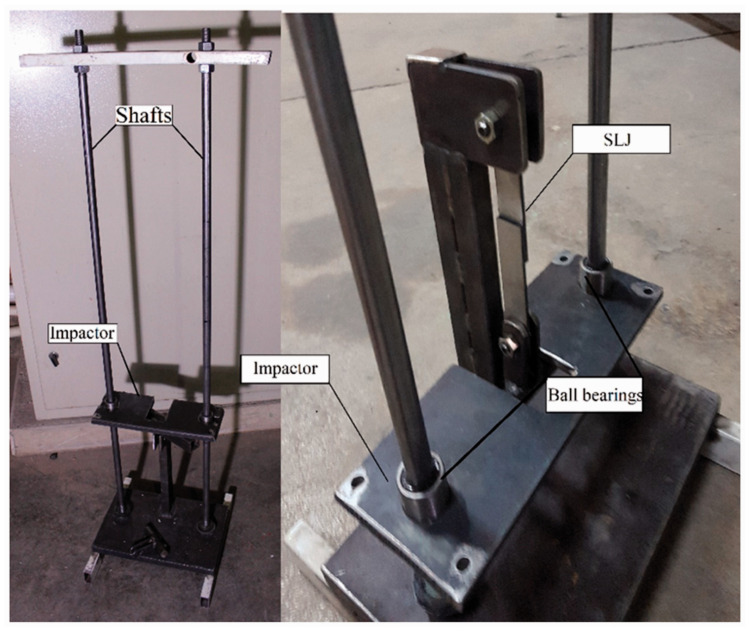
The device used to apply the impact loads (already developed and used by authors [13]).

For the impact fatigue analysis, in this study, similar (steel/steel) and dissimilar (composite/steel) adhesive joints were subjected to various levels of low energy impacts, and the corresponding lives were analyzed. Then, by using the bi-adhesive technique, the impact fatigue life of the same joints was improved. The rate of improvement in fatigue life using the bi-adhesive technique was analyzed experimentally. In order to construct the J-N curves, joints were tested at different impact energy levels. In order to apply the cyclic low-energy impact fatigue loads, an in-house drop weight low energy impact test device with a maximum capacity of 50 N.m already designed and employed in previous studies [13,20] was used in the current research (see Figure 3). The impactor weight was set to 5 kg, and by changing the impactor height, the joints were subjected to different levels of impact energy. By using the low-energy impact test machine, joints were subjected to different levels of impact loads (5 to 15 N.m). Due to the large mass used (5 kg) and also the low impact energy considered, the mass was dropped from a very low height resulting in a low impact velocity. Although, in this condition, a small rebound is assumed, the influence of the rebound speed was considered negligible for all tested joints. Figure 3 shows the device used to apply the impact fatigue loads.

Impact loads were repeated until joint failure. Three samples were tested for each condition. The impact energy level was defined by the height of the impactor that was controlled using a vertical guide. In order to minimize friction between the impactor and the guide shafts, lubricated ball bearings were used. Accordingly, the effect of friction was considered negligible. The ball bearings can also align the impactor during the test. As also mentioned before, pins were used to mount the joints in the impact test machine. The impactor applies the impact loads to the joints through the pins. The energy absorbed by joints is the sum of the energy applied until the failure of SLJs. Consequently, it is simply calculated from the energy of each impact multiplied by the number of impact cycles to the failure of the joint.

### 2.5. Finite Element Analysis (FEA)

**Figure 4:**
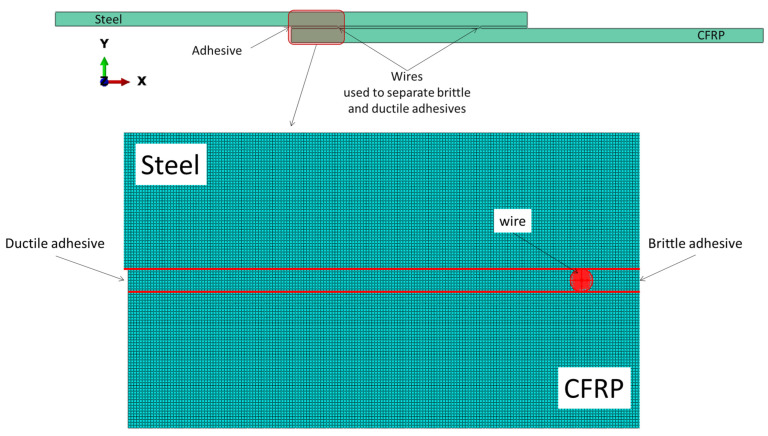
Simulated SLJ and the considered mesh configurations.

The stress distribution across the bondline in a bi-adhesive joint is significantly influenced by the presence of a ductile adhesive at the bonding ends. In order to obtain a better insight into the stress state and also to find a possible relationship between the stress distribution and the obtained experimental data, a simple linear elastic FEA was performed for joints with different substrates and different length ratios of the adhesives. The same elastic properties as given in the previous section were used. The linear FE analysis allows only to have an idea of the ratio between the stress in the two adhesives and the stress distribution. However, considerations of the failure modes and failure mechanisms are not possible with this analysis. A 2D analysis was performed using Abaqus. Eight elements were created along the adhesive thickness. Figure 4 shows the meshed FE model. The samples were meshed by a four-node bilinear plane strain quadrilateral, reduced integration elements. Aluminum wires used in experiments to separate the adhesives were also considered in the numerical simulation, as shown in Figure 4. A displacement of 0.5 mm was applied to one end while the other end of the joint was clamped. The peel stress along the mid-plane of the adhesive layer was analyzed in this study.

## 3. Results and Discussion

### 3.1. Static Tests

**Figure 5:**
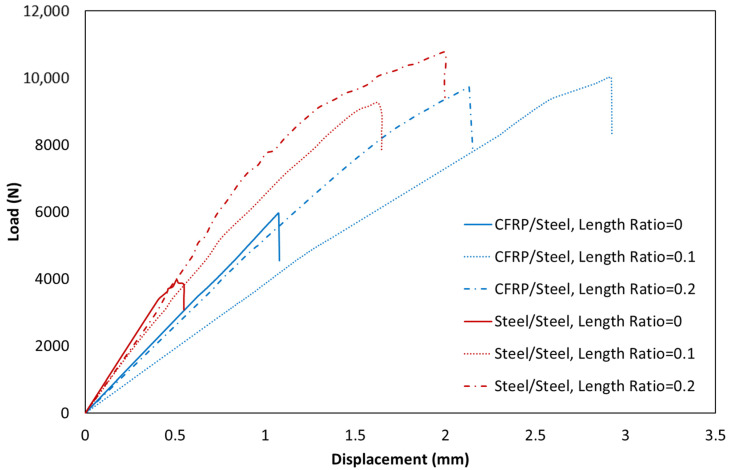
Load–displacement curve of similar (steel/steel) and dissimilar (CFRP/steel) joints under static loading conditions.

Figure 5 shows the typical behavior of various joints with different substrates and different adhesive length ratios under static loading conditions. First, it was experimentally shown that the bi-adhesive technique could significantly improve the static strength of the joints. The static strength test results also show that the optimal length ratio is influenced by the assembly configuration. Based on the results, the length ratio that leads to the maximum strength is different for dissimilar and similar SLJs. In joints with similar adherends, the best static strength was obtained for a length ratio of 0.2, while the best improvements for composite-steel joints were related to a length ratio of 0.1. As presented in Figure 5, the effects of length ratio are more significant in joints with similar adherends than in dissimilar ones. It should be noted that the displacement shown in Figure 5 is the displacement of the crosshead and does not accurately reflect the displacement that the bondline experienced during the test.

One of the key factors in manufacturing bi-adhesive joints is to find the optimum length ratio. It is obvious that the optimum length ratio depends on the joint geometry and the stiffness of the adherends.

In the case of dissimilar joints, the symmetry of the stress along the overlap would be lost due to the different stiffness of the substrates. Therefore, the edge effects are more critical at the edge with the lower stiffness substrate. Less stiffness causes more rotation of the substrate during the test, resulting in greater peel stress in the adhesive layer. It is obvious that the failure starts from the critical points. Another failure mechanism is delamination, which is a common problem in composite laminates and has been studied by several authors [27,28,29].

**Figure 6:**
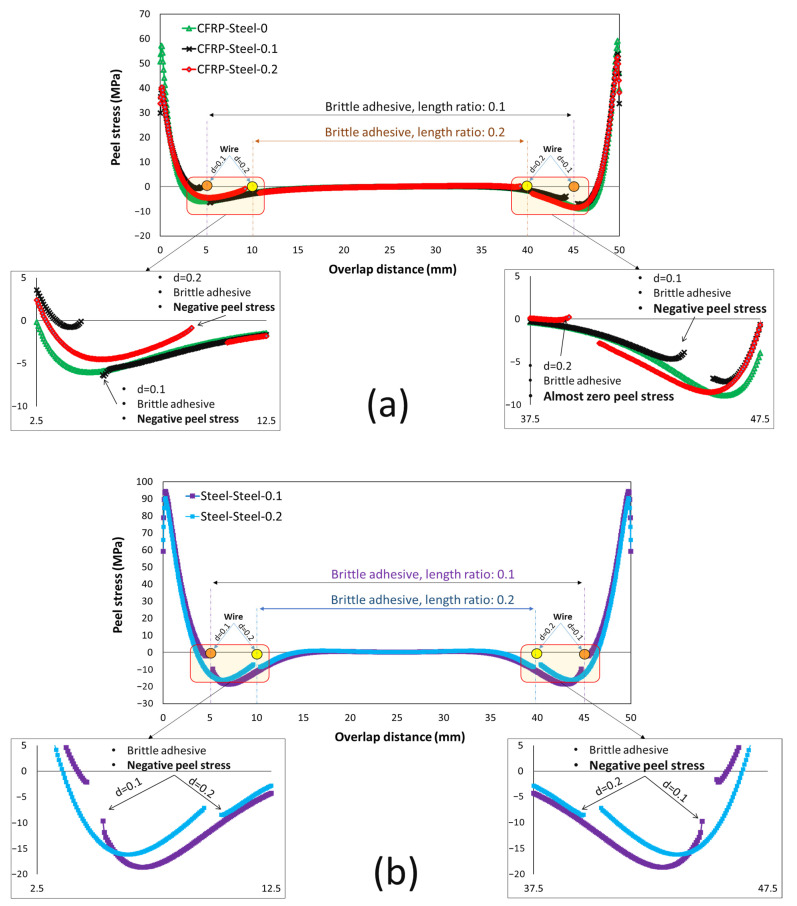
Normal stress distribution along the bondline in the tested bi-adhesive SLJs (**a**) dissimilar CFRP-Steel and (**b**) similar steel joints.

For joints with a single brittle adhesive, a significant stress concentration is observed at the lap ends, and failure is mainly driven by crack initiation at the ends of the bondline. In bi-adhesive joints, where part of the brittle adhesive is replaced by a ductile adhesive at both ends of the overlap, not only does the stress concentration at the lap ends disappear (due to the plastic deformation of the ductile adhesive), the brittle adhesive at its ends experiences compressive (negative) peel stresses, which is one of the main reasons behind the significant improvement in the static strength of bonded bi adhesive joints compared to single adhesive SLJs. Figure 6 shows the distribution of the peel stress along the single- and bi-adhesive layer in dissimilar (Figure 6a) and similar (Figure 6b) adherend SLJ subjected to 0.5 mm elongation. Due to the different properties of the substrate, a non-symmetric stress distribution is observed along the overlap (Figure 6a), while for similar joints, the stress distribution is symmetric (Figure 6b). However, for the analyzed joints, the brittle adhesive experiences high peel stress at the bonding ends (around 60–70 MPa in the case of the linear elastic assumption), while the stress at the tip of the brittle adhesive reduces to compressive loads where the normal stress applied to the brittle adhesive is no longer peeling stress and varies between 0 and −17 MPa.

### 3.2. Cyclic Impact Analysis

One of the most destructive loads that joints can be subjected to in service is cyclic impact, which can lead to unpredictable catastrophic failure. Although the energy applied during each impact cycle is much less than the impact strength of the joints, significant damage occurs due to the stress waves propagating through the adhesive layer that accumulates damage in the adhesive layer during impact fatigue. An increasing number of impacts leads to a gradual deterioration of the mechanical properties of the adhesive on the one hand and an accumulation of microcracks/damage on the other. Accordingly, the ability of the adhesive to dampen the stress waves would be significantly reduced after a certain number of impact cycles [13].

### 3.3. J-N Behavior

Designing a durable adhesive bond is the most important end goal when designing bonded assemblies. A key factor in achieving this is to make the stress more uniform along the bondline. As previously discussed, the technique of the bi-adhesive, or more generally graded adhesive, is one solution to achieve this goal. The length ratio of the adhesives, as well as the stiffness of the substrate, play a key role in reducing local stresses in SLJs. In order to investigate the impact of these parameters on the impact fatigue life of different SLJs, cyclic impact tests were performed at three different impact energy levels. In order to analyze the impact fatigue behavior of the joints, a J-N methodology is used in which the impact fatigue life of the joints is plotted against the applied impact energy per cycle in a semi-logarithmic plot.

**Figure 7:**
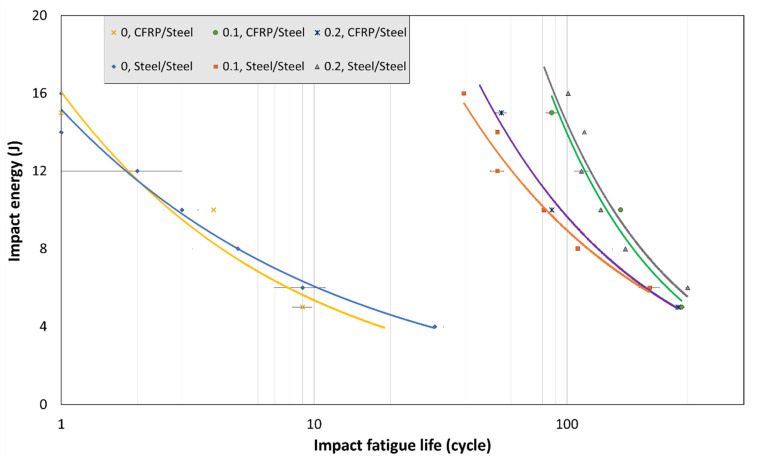
J-N response of similar and dissimilar bi-adhesive single lap joints subjected to cyclic impact loads.

By using Basquin’s type equation, a logarithmic trend line was fitted to the experimental data to estimate the cyclic life of the joints at lower impact energy levels corresponding to higher impact fatigue lives. Figure 7 shows the J-N behavior of the joints for different length ratios in the tested dissimilar steel-to-CFRP bonded joints and also for similar steel-to-steel bonded configurations.

The bi-adhesive technique was able to significantly improve the impact fatigue life of the tested samples. It was found that for dissimilar joints, increasing the length ratio from 0.1 to 0.2 reduced the impact fatigue life, while for similar steel joints, the length ratio of 0.2 resulted in the highest impact fatigue lives. A possible reason behind this difference is the different peel stress distributions that the bondline experiences in different joints. Based on the numerical analysis results shown in Figure 6, in CFRP/steel joints, the brittle adhesive at its end experiences a higher compressive load for a 0.1 length ratio than for a 0.2 length ratio. On the other hand, for steel/steel joints, the normal compressive stresses applied to the ends of the brittle adhesive are higher for joints with a length ratio of 0.2 (see Figure 6b). However, under all conditions tested, an impressive increase in impact fatigue life was observed for bi-adhesive joints compared to single-rigid adhesive joints.

As can be clearly seen in Figure 7, no impact fatigue endurance limit can be determined for the tested joints. The same trend was noted in previous studies [13]. However, still, further research is needed for very low-impact energy levels to investigate the fatigue strength of joints subjected to very high cyclic impacts. Based on the results shown in Figure 7, in case of any possible endurance limit, it would be much lower than the impact strength of the joints (less than 4 N.m for the tested joints). The endurance limit in impact fatigue is also much lower than normal fatigue [15]. Thus, it can be said that the impact fatigue loading regime has a greater potential to cause premature failure of bonded structures. The experiments proved that increasing the impact energy significantly decreased the impact life of the joints. As expected, it was found that joint life is not a linear function of impact energy; for example, a 50% reduction in impact energy increases fatigue life by more than 70%.

The curves fitted to the experimental data points (Figure 7) also showed that the effects of length ratio become more pronounced with increasing impact energy. On the other hand, it should be noted that the optimal length ratio is also a function of the ratio of Young’s modulus of the two ductile and brittle adhesives [18,21]. It should be noted that due to the viscoelastic behavior of the adhesives, increasing the strain rate decreases the ductility of the adhesives resulting in a different E ratio and consequently leading to a different optimal length ratio. As already shown in [20], the ductile adhesive is more sensitive to the loading speed than the brittle adhesive for the tested materials. Therefore, the E-ratio would increase as the load rate increased. Accordingly, the optimum length ratio for quasi-static testing is not necessarily an optimum ratio for high strain rate and impact loading conditions. Not only the loading speed but also the rigidity of the substrate changes the optimal length ratio. Using different materials as substrates also complicates the problem due to the non-symmetrical distribution of the peel stress along the bondline. A nonuniform stress distribution along the bondline also changes the optimum length ratio. Based on the results obtained, the best length ratio for dissimilar joints is 0.1 (among the tested conditions), while for similar steel/steel SLJ is 0.2. Another reason behind this difference is the different peel stress state at the boundary of the two adhesives that was discussed earlier.

However, to analyze the sensitivity of each parameter (substrate stiffness, dissimilarity of bonded substrates, and loading rate), further experiments are needed, where all these parameters need to be experimentally analyzed at multiple levels.

### 3.4. Total Energy Absorption

**Figure 8:**
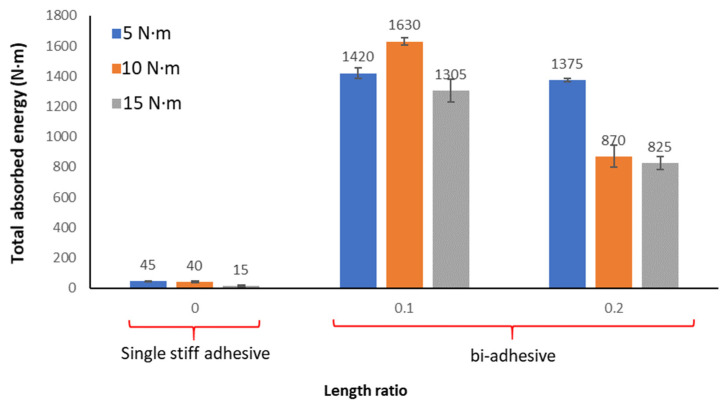
Total energy absorption of single- and bi-adhesive CFRP/steel joints subjected to different impact energy levels.

In this analysis, the total energy absorption is defined as the summation of the energy absorbed during the impact loads until failure. The energy applied at each impact is calculated based on the falling height and the weight of the impactor. According to the results, the bi-adhesive technique shows an impressive increase in the energy absorption capacity of the tested dissimilar joints (as shown in Figure 8). According to the results, the impact energy absorption for joints subjected to 10 N.m impact loads increases from 40 N.m for the length ratio of 0 (single brittle adhesive joints) to 1630 and 870 N.m for bi-adhesive joints with the length ratios of 0.1 and 0.2, respectively. It means that besides the increase in the static strength, the bi-adhesive technique increased the impact energy absorption capacity by a factor of around 41, which is a significant improvement in the damping capacity of the tested CFRP/steel bonded joints.

Energy absorption is a function of the number of impact cycles and the energy level of each impact. The results show that for joints subjected to an impact load of 10 N.m, the total energy absorption is higher than for joints subjected to a fatigue impact load of 5 and 15 N.m. The total energy absorption not only influences the applied impact load but is also affected by the adhesive length ratio in bi-adhesive joints. Increasing the impact energy from 5 to 10 decreases the total energy absorbed for joints with a length ratio of 0.2, while it is the opposite for a length ratio of 0.1. Therefore, to achieve an optimum condition with maximum cyclic impact energy absorption, the interaction between the level of impact loading and the load ratio of the adhesive should be analyzed.

### 3.5. Fracture Surface Analysis

**Figure 9:**
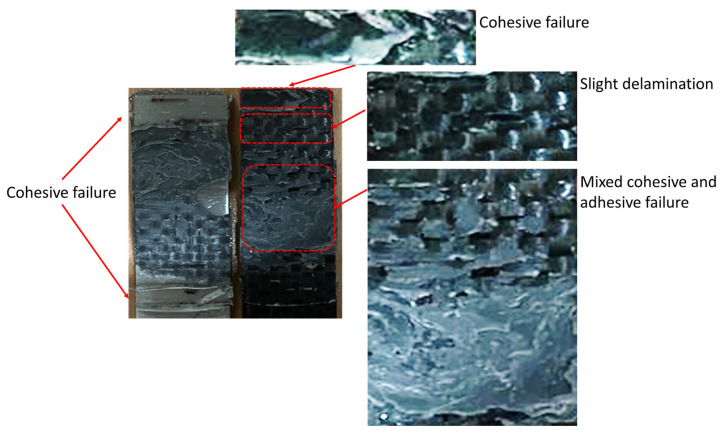
Schematic crack path and a typical fracture surface of the tested dissimilar SLJs.

One of the key parameters to find the root cause of failure is fractography. As discussed in [13], for similar steel substrates bonded with a single stiff/brittle adhesive subjected to impact fatigue loading, failure begins in the middle of the overlap. It was found that at lower energy levels, the cracks are more concentrated in the center of the bondline, while at higher impact energies, the cracks are more widely spaced along the overlap. It is also clear that by increasing the number of impacts, the damage propagates and causes a weakening of the joint. Failure occurs when the damaged area reaches a critical level where the remaining impact strength of the joint is less than the applied impact fatigue load. Although a similar failure initiation and propagation can be assumed for CFRP/steel joints with a single brittle adhesive, however, the failure mechanism in dissimilar CFRP-steel SLJs with two different adhesives used along the lap is much more complex and, as shown in Figure 9, a combination of delamination in the CFRP part, zones with interfacial failure between the adhesive and the substrates, and a region of cohesive failure mode. Since two different adhesives with different behavior were used along the overlap, a combination of ductile damage and brittle fracture is to be expected before the failure of the joint. On the other hand, impact fatigue cycles can also produce local damage and microcracks. The damage accumulation and the growth of the damage zones finally led to the final failure of the tested bi-adhesive connections. Local delamination was also observed in composite substrates. Although the impact fatigue shows distinct zones along the overlap for single adhesive SLJs (see [13] for more details), these distinct zones were not clearly observed on the surface of bi-adhesive joints. Due to the stress wave distribution along the bonded area [20], a fatigue damage zone is expected in the area with the brittle adhesive, while the ductile adhesive is usually less sensitive to the applied low-energy impact loads due to the higher damping capacity. As shown in Figure 9, the fracture process of the bi-adhesive SLJs is a combination of cohesive failure through the adhesive layer, adhesive failure, and a local CFRP delamination.

## 4. Conclusions

In this study, the impact fatigue life of different adhesive joints was first analyzed experimentally. Subsequently, the fatigue strength of the CFRP/steel joints was then improved using the bi-adhesive technique. By using FEA, the possible reasons behind the impressive improvement in the static strength and impact fatigue life of bi-adhesive joints compared to single-adhesive joints were analyzed. The results showed that the impact fatigue life of the bonded joints could be greatly improved (by a factor of 41 in some cases) by using two adhesives along a single overlap. However, it should be noted that changing the length ratio (d) of the adhesives can significantly alter the results. The results showed that the optimal length ratio is a function of the substrate materials. For joints with similar substrates, the length ratio of 0.2 resulted in higher cycle fatigue, while for dissimilar CFRP/steel SLJs, the 0.1 length ratio resulted in the best strength. The possible reason for this difference is the difference in compressive stresses that brittle adhesive experiences at its ends in bi-adhesive joints. Based on the results, the use of bi-adhesives instead of a single adhesive along a single overlap for joints subjected to impact fatigue loading is recommended. However, the effects of different parameters, such as strain rate, substrate materials, and adhesive properties, should be considered to define the best length ratio. Although no clear fatigue limit was observed for the tested joints, if a fatigue limit for impact strength is assumed, it would be much lower than the impact strength of the joints.

## Figures and Tables

**Table 1:** Mechanical properties of the adherends [20,22].

CFRP	Steel	
--	215	Yield Stress (MPa)
E1 = E3 = 82 E2 = 10, G12 = 3 *	197	Modulus of elasticity (GPa)
0.24	0.29	Poisson’s ratio
620 (along directions 1 and 3, see Figure 1)	505 (UTS)	Tensile strength (MPa)

* 1, 2, and 3 are the directions along the length, thickness, and width of the specimen (see Figure 1).

**Table 2:** Properties of the considered epoxy adhesives [20].

Properties	Brittle Adhesive	Ductile Adhesive	Unit
Modulus of Elasticity	2010 ± 150	980 ± 117	MPa
Elongation	1.4 ± 0.2	33 ± 4.2	%
Poisson’s ratio	0.31 ± 0.02	0.39 ± 0.03	-
Gel time *	5	60	min
Curing temperature *	25	25	°C
Curing time *	4	7	Days

* Based on the manufacturer data.

## Data Availability

Data is contained within the article.
